# Assessment of the Management of Patients with Chronic Pain Referred to a Specialized Pain Unit: A Cross-Sectional Multicenter Study (the DUO Project)

**DOI:** 10.3390/jcm11133586

**Published:** 2022-06-22

**Authors:** Víctor Mayoral Rojals, Ángeles Canós Verdecho, Begoña Soler López, the Team DUO

**Affiliations:** 1 Pain Unit, Hospital Universitari de Bellvitge, IDIBELL, L’Hospitalet de Llobregat, 08907 Barcelona, Spain; victormayoral@mac.com; 2 Pain Unit, Hospital Universitari i Politécnic La Fe, 46026 Valencia, Spain; canos_marver@gva.es; 3 Medical Department, E-C-BIO, S.L., Las Rozas, 28230 Madrid, Spain

**Keywords:** chronic pain, pain unit, quality indicators, analgesics, opioids, gender differences

## Abstract

A multicenter cross-sectional study was designed to assess the quality of treatment of 1190 patients with chronic pain at the time of referral to a specialized pain unit. A total of 119 physicians from 77 pain units throughout Spain collected 23 indicators of the quality of care from 10 consecutive clinical records of chronic pain patients (5 men, 5 women). Degenerative spinal diseases (38.6%) and lumbosciatic pain (29.8%) were the most common etiologies. At the time of referral to the pain unit, 9.8% of patients were not receiving any analgesic treatment. Treatment was modified in 88.1% of the patients by adding adjuvant drugs, adding opioids or increasing the doses of analgesic medications, and using analgesic techniques. Women had higher percentages of osteoarthritis, headache and fibromyalgia as the cause of pain, longer duration of pain and severe pain intensity, and a higher proportion of changes in the diagnosis of the underlying condition with which they had been referred to the pain unit. Improvements should be made in the patient management and referral protocols not only in the clinics prior to patient referral to the pain unit, but also in the pain units themselves.

## 1. Introduction

Chronic pain is one of the most common reasons for adults to seek medical care, particularly in primary care [1], and is associated with restrictions in daily life activities and mobility, reduced quality of life, anxiety and depression, and dependency on opioids [2,3]. The high prevalence of chronic pain and pain-related diseases as the leading cause of disability and disease burden globally has been confirmed in different studies, where 15–25% of adult population suffer chronic pain, reaching to the 50% in older than 65 [2,4,5,6]. Low back pain and recurrent tension-type headache are the conditions that cause most disability and dysfunction [7]. Additionally, the demand for pain care shows an increasing trend due to aging of the population and a high prevalence of chronic diseases [8,9].

Despite increased focus on the importance of pain control and effective analgesic medications, inadequate pain management has been widely reported with a large variability of undertreatment across studies and settings [10,11,12]. Barriers to the implementation of adequate pain control are multifactorial involving patient-related and biopsychosocial factors, disease-related factors, underestimation of pain intensity, lack of adequate training of healthcare providers, inadequate pain evaluation, and especially the complexity of the pathophysiological mechanisms of pain [13,14,15,16]. A multidisciplinary approach to pain management based on early multidimensional diagnosis of chronic pain and rapid initiation of evidence-based therapy according to an individual treatment plan is necessary to ensure the best outcomes [17,18].

Pain units were created to provide multidisciplinary pain assessment and care, involving a team of anesthetists, neurologists, psychiatrists, occupational therapists, nursing staff, and rehabilitation physicians [12]. In a meta-analysis of 65 studies, the effects of multidisciplinary treatments for chronic pain appeared to be stable over time and were not limited to improvements in pain but also extended to mood, interference with daily activities, and behavioral variables such as return to work or use of the health care system [19]. Multimodal pain management directed by pain specialists in pain clinics plays a pivotal role in the care of patients with chronic pain [20,21,22,23,24,25,26], but there is still little information on different aspects related to the profile of patients referred to these units, reason for referral, characteristics of pain management before referral, interventions and treatment prescribed by pain unit specialists, and outcomes attained. Although the use of the pain units is recommended worldwide, local rules for the referral of the patients could influence the characteristics of the patients for the first referral. Thus, in Spain, patients may be referred to the pain unit from primary health care or a specialist, mainly outpatients, when there is a problem diagnosing the underlying disease that generates the pain, and when the pain has not been controlled. Therefore, it is to be expected that the patient referred to the pain unit does not have the pain controlled, and this should be the main reason for his consultation [26]. This study aimed to evaluate the current practice of patients with chronic pain referred to pain units from a national perspective, and to provide useful information to assist stakeholders involved in pain care in their decision-making challenges.

## 2. Materials and Methods

### 2.1. Design

This was a multicenter, cross-sectional study conducted in pain units throughout Spain over a 5-month period (May–September 2020) and based on data collected from the medical records of patients with chronic pain (the DUO project). DUO is the Spanish acronym of “Dolor y Uso de Opiáceos” (Pain and Opioids Use). The primary objective of the study was to assess the clinical condition of patients with chronic pain at the time of referral to the pain unit, including the control of pain and details of treatment (medication and doses). The secondary objective was to assess the management of patients in the pain unit. The study protocol was approved by the Clinical Research Ethics Committee (CEIC) of Hospital Universitari de Bellvitge (code PR048/20, approval date 27 February 2020), and informed consent was waived because study data were collected from the electronic patients’ medical records. All data were anonymized.

### 2.2. Participants

Patients of both genders aged 18 years or older were eligible provided that they presented with chronic pain, had been managed in the outpatient setting, and were referred to the pain unit for the first time. Chronic pain was defined according to the International Association for the Study of Pain (IASP) as either chronic primary or secondary pain in ≥ 1 anatomic region that is either persistent or recurs for >3 months and causes functional disability and emotional stress [24]. Chronic primary pain syndrome was also defined as pain that cannot be accounted for by any other chronic pain disorder.

### 2.3. Study Procedures

The scientific committee of the study was composed by two pain management specialists (V.M.R., A.C.V.) who developed the study questionnaire based on different recommendations of clinical practice guidelines for pain management [25,26]. The questionnaire included 35 questions, which were grouped into three sections: (a) indicators of the participating physician (4 items), (b) indicators of structure of the pain unit (8 items), and (c) indicators of the chronic pain process (23 items). The description of the study questionnaire is included in the Supplementary Material.

Study participants were staff physicians working in pain units throughout Spain. They were recruited by the contract research organization through formal e-mail invitations that included a brochure with full information about the project. The questionnaire was lodged in an internet microsite that could be accessed via a weblink, and only physicians who accepted to participate in the study were provided with access to the questionnaire platform URL and the user’s password. Participation in the study was anonymous and voluntary. To complete the section of process indicators, participating physicians collected data of 10 consecutive patients (5 men and 5 women) with chronic pain who were referred to the pain unit during the study period.

### 2.4. Statistical Analysis

A sample size of 120 pain units was necessary to describe the process indicators selected for the study with an estimated precision of ±6% in the confidence intervals (CI) of the proportions, with a 94% statistical power for a two-tailed alpha error of 0.05. Categorical variables are expressed as frequencies and percentages, and continuous variables as mean and 95% CI. The chi-square test or the Fisher’s exact test was used for the comparison of categorical variables, and the Student’s *t*-test for quantitative variables. In relation to process indicators, differences between men and women were analyzed using the Student’s *t*-test or the Mann–Whitney *U* test for the comparison of the mean values and 95% CI for valid cases (number of patients in which the value of the variable was available) and the mean percentages and 95% CI of compliance with the indicator for valid cases in each pain unit. Statistical significance was set at *p* < 0.05. The SPSS version 27.0 statistical package (IBM SPSS, Chicago, IL, USA) was used for the analysis of data.

## 3. Results

### 3.1. Characteristics of Participants

A total of 119 physicians from pain units completed the study questionnaire and provided pooled data of 1190 patients with chronic pain. Pain units were located in 16 out of total 17 regions of Spain. There were 57 men and 62 women, with a mean age of 46.2 years and mean years of professional experience of 12.5. Most physicians were specialists in anesthesiology (86.6%) and worked in public hospitals (89.9%) (Table 1).

### 3.2. Structure Indicators

The mean number of new patients referred to the pain unit in one month was 104, accounting for 26.2% of new consultations. Pain assessment protocols were available in 78.8% of the pain units, and validated scales for the assessment of chronic pain were used in 95.8% of the cases. Validated scales for assessing quality of life and mental health were used in 50.4% and 28.6% of cases, respectively (Table 1).

### 3.3. Process Indicators

Details of process indicators are shown in Table 2 and Table 3. Around 45% of patients were referred to the pain unit from orthopaedic surgery and traumatology; men as compared with women were more frequently referred from neurology/neurosurgery (11.7% vs. 7.6%, *p* = 0.02) and oncology (4.2% vs. 2.4%, *p* = 0.03) services, whereas a higher percentage of women were referred from rheumatology services (9.3% vs. 4.7%, *p* = 0.001). Women were also older than men (61.9 vs. 59.1 years, *p* = 0.001). Regarding the employment status, more men were in the active category, whereas more women were housewives. Leading causes of chronic pain were degenerative spinal diseases and lumbosciatic pain. Lumbosciatic pain was significantly more common in men (*p* = 0.001), whereas osteoarthritis (*p* = 0.001), headache (*p* = 0.033) and fibromyalgia (*p* < 0.0001) were significantly more frequent in women. Women also showed a significantly longer duration of chronic pain (32.7 months) than men (26.8 months) (*p* = 0.007). In relation to the type of pain, significant gender differences included somatic pain (*p* = 0.001) and primary pain (*p* = 0.045) more frequent in women, and neuropathic pain more frequent in men (*p* = 0.008). The intensity of pain assessed on a 0–10 scale at the time of pain unit consultation was 6.9 (95% CI 6.7–7.2) in women and 6.6 (95% CI 6.3–6.8) in men (*p* = 0.041). Moreover, severe pain was reported by a higher percentage of women (*p* = 0.033) and mild pain by a higher percentage of men (*p* = 0.033).

More than 80% of patients had impaired functionality due to chronic pain and more than 50% sleep disturbances caused by the pain condition. Breakthrough pain was present in 27% of patients. Differences between men and women in these variables were not observed.

**Figure 1:**
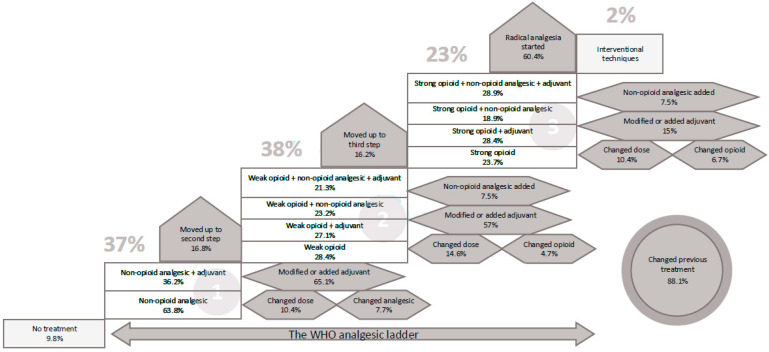
Treatment details according to the steps of the analgesic ladder in first-time referral to the pain unit (white boxes) and actions taken at the pain unit regarding modification of treatment (grey boxes).

Figure 1 summarizes treatment according to the analgesic ladder steps on referral to the pain unit and modification of treatment recommended in the pain unit. A total of 9.8% of the patients with chronic pain were not receiving any treatment at the time of referral to the pain unit. The remaining 90.2% were receiving some analgesic treatment, with 37.8% of patients treated according to the first ladder step (mainly non-opioid analgesics only), 38% to the second ladder step (weak opioids with or without adjuvant drugs in 21% of cases), and 23% to the third step (strong opioids alone in only 5.5% of cases) (Table 2). Other therapeutic interventions, such as peripheral nerve block, electrical stimulation techniques, analgesia through the spinal route, sympathetic/neurolytic block were only used in 2% of patients. On the other hand, only 44% of patients had received instructions for use of rescue analgesia. Gender-related differences in the distribution of patients according to the analgesic ladder were not observed.

Paracetamol, non-steroidal anti-inflammatory drugs (NSAIDs), tramadol, metamizole and venlafaxine were the most commonly used medications (Table 3). Metamizole was use by a significantly higher percentage of women as compared with men (28.2% vs. 22%, *p* = 0.042), whereas NSAIDs were more commonly used in men (42.6% vs. 34.7%, *p* = 0.049). On admission to the pain unit, drug treatment was changed in 88.1% of patients, with lack of efficacy being the main reason for change in about 60% of patients. Addition of one or more adjuvant drugs, modification of doses of second step analgesics, change from the first to the second step, and change from weak to strong opioid were the actions more frequently recommended, with similar percentages in men and women (Table 3). However, the previous diagnosis was modified in 19% of women and in 14.8% in men (*p* = 0.043).

Finally, after the first consultation in the pain unit, 90.3% of patients were appointed for a second visit in the pain unit, 4.6% were referred to the primary care setting, 2.8% to the same service or specialty than the one they came from, and 2.6% to a different specialty.

## 4. Discussion

This study aimed to gather knowledge on the clinical profile of patients with chronic pain at the time of referral to a specialized pain unit, usually because of poor control of pain. The nationwide perspective of the project is supported by the participation of 119 physicians from 77 pain units out of the 123 accredited units in the Spanish public healthcare system [27]. Additionally, the physicians who completed the survey had a mean experience in the pain field of 12 years excluding their training period, and most of them were specialists in anesthesiology. Seventy-nine percent of pain units had a written protocol for pain assessment, although it would be desirable for all units to implement a protocolized evaluation of pain. There was a reduced percentage of units in which health-related quality of life and mental health of patients with chronic pain were evaluated on a routine basis, a relevant aspect that should be improved because of the deleterious effect of chronic pain on daily functioning and the risk of triggering anxiety, depression, and other mental health issues [28]. Perhaps one of the barriers of the pain therapy centers is that they have limited time and insufficient resources to test and follow patients’ quality of life.

Patients were referred to the pain units from different settings, especially from orthopaedic surgery and traumatology (45%), followed by primary care (14%) and physical medicine and rehabilitation (13%), which is consistent with common causes of pain, including degenerative spinal diseases, lumbosciatic pain, and arthrosis. In a previous study of 269 patients referred to 12 outpatient hospital pain clinics in Catalonia, Spain, 50% of patients were referred by specialists in orthopaedic surgery and traumatology and 20% by primary care [21]. Despite that fact that most patients suffered from moderate-severe pain with impaired functionality in more than 80% of cases and sleep disturbance in almost 60%, pain had not been measured using a validated instrument in 40% of patients prior to referral to the pain unit. The impact of pain on the quality of sleep was only evaluated in 16% of patients. Moreover, chronic pain was long-lasting with a mean duration of more than 2 years. These findings indicate that there is still large room for improvement in the management of chronic pain before referral to pain specialists. However, differences in the duration of pain according to types and causes of pain or referral services were not analyzed. Some differences found between men and women could be expected as women showed a higher proportion of arthrosis, headache and fibromyalgia, as well as pain intensity, which agrees with data from a Norwegian population-based study in which women reported significantly higher pain intensity scores than men [29].

Data reported in other studies regarding undertreatment of chronic pain [12,21,30,31] were also found in the present study, including around 10% of patients who had not received any pain therapy and the fact that drug treatment was modified in 88.1% of patients on the first visit to the pain clinic. The proportion of patients receiving drugs of the first and second step of the WHO analgesic ladder was similar (38%), and 23% of patients were treated with strong opioids (third step). It should be noted that 45.4% of patients received adjuvant drugs, particularly over the first and second steps. The most commonly prescribed analgesic medication was paracetamol (in more than half of the patients) followed by tramadol, NSAIDs, and metamizole. However, paracetamol has been shown to be ineffective in the treatment of low back pain and provides minimal short-term benefits in patients with osteoarthritis [32]. The use of adjuvant agents together with the main drugs is allowed on all analgesic ladders [33]. Adjuvant drugs improve the analgesic response and are particularly useful for some types of pain, such as neuropathic pain. These drugs include antidepressants, anxiolytics, steroids, muscle relaxants, capsaicin or local anesthetics. The analgesic effect is probably produced via enhancement of transmitter concentrations in pain-modulating pathways [34]. Interestingly, venlafaxine was the most common adjuvant drug used by patients at the time of referral to the pain unit (26.6%), but duloxetine, which has significant analgesic effects for managing chronic pain associated with fibromyalgia and peripheral neuropathic pain [35], was used by 1.8% of the patients only.

The main action taken in the pain unit was a change of treatment in 88.1% of the patients to achieve a better control of pain because of lack of efficacy or insufficient doses of previous medications. In patients in the second and third steps, a non-opioid analgesic was added. Moreover, 17% of patients in the first analgesic step moved to the second step, and 16% of those in the second step moved to the third step. Interventional procedures for analgesia are measures mainly adopted by pain specialists and were indicated in 60% of the patients. In none of the actions taken at the pain unit, significant differences between men and women were found, except for modifying the previous diagnosis, which occurred more frequently in women than in men, although changes related to individual diagnosis were not evaluated. On the other hand, some of the differences found in our study between men and women in relation to higher pain intensity, longer duration of pain, and chronic conditions, such as headache, back pain, and fibromyalgia, have been reported in other studies also [29,36].

Pooled data collection prevented individual patient comparisons. Data were recorded during the first visit to the pain clinic and, although pain conditions continued to be managed by pain specialists in subsequent visits, the course of patients was not evaluated. Despite these limitations, the sample of pain units accounted for 63% of all pain units available in the public healthcare system of the country, supporting the representativeness of the sample.

## 5. Conclusions

The present findings indicate that improvements should be made in the patient management and referral protocols, with reinforcement of the importance of using validated instruments to assess the intensity of pain and the impacts of chronic pain on quality of life and mental health. Gender-related differences require attention, especially in relation to higher pain intensity and causes of chronic pain in women. Efforts should be made to provide an integrated multidisciplinary care of patients with chronic pain with the objective of optimizing drug treatment and improving adequate long-lasting control of pain.

## Figures and Tables

**Table 1:** Characteristics of 119 participants.

Variables	Number (%)	Mean (95% CI)
Participating physicians		
Gender		
Male	57 (47.9)	
Female	62 (52.1)	
Age of the participating physician		46.2 (44.5–48.0)
Years of professional experience in the field of pain		12.5 (10.8–14.1)
Current clinical specialty		
Anesthesiology	103 (86.6)	
Primary care	5 (4.2)	
Physical medicine and rehabilitation	10 (8.4)	
Neurosurgery	1 (0.8)	
Structure indicators		
Type of center to which the unit belongs		
Public hospital/center	107 (89.9)	
Mixed center	12 (10.1)	
What level does your pain unit correspond to?		
Level I	10 (8.4)	
Level I	58 (47.1)	
Level III	53 (44.5)	
Number of patients seen in the unit in one month		462.6 (402.8–522.4)
Number of new patients referred to the unit in one month		103.8 (89.8–117.8)
Does the unit have a pain assessment protocol?		
No	25 (21.2)	
Yes	93 (78.8)	
Does the unit use validated chronic pain assessment scales?		
No	5 (4.2)	
Yes	114 (95.8)	
Is health-related quality of life of patients with chronic pain evaluated using validated scales?		
No	59 (49.6)	
Yes	60 (50.4)	
Is mental health of patients with chronic pain evaluated using validated scales?		
No	85 (71.4)	
Yes	34 (28.6)	

CI: confidence interval; Level I: monographic unit; Level II: multidisciplinary pain treatment unit; Level III: multidisciplinary pain center or unit for the study and treatment of pain.

**Table 2:** Process indicators in 1190 patients with chronic pain referred to the pain unit.

Variables	Total % (95% CI)	Gender		*p* Value
		Men % (95% CI)	Women % (95% CI)	
Specialty from which the patient is referred				
Orthopaedic surgery and traumatology	44.7 (40.6–48.9)	43.5 (38.6–48.3)	45.8 (41.0–50.6)	
Neurology/neurosurgery	9.7 (7.3–12.0)	11.7 (8.5–14.9)	7.6 (5.1–10.1)	0.02
Rheumatology	7.0 (5.3–8.7)	4.7 (2.8–6.5)	9.3 (6.8–11.9)	0.001
Primary care	14.3 (11.3–17.3)	13.8 (10.6–17.0)	14.9 (11.3–18.5)	
Oncology	3.3 (2.1–4.5)	4.2 (2.7–5.8)	2.4 (1.1–3.7)	0.03
Physical medicine and rehabilitation	13.0 (10.0–15.9)	12.7 (9.4–16.1)	13.2 (9.5–16.9)	
Internal medicine	1.4 (0.7–2.2)	1.7 (0.7–2.7)	1.2 (0.3–2.1)	
Others	7.0 (5.2–8.8)	7.7 (5.1–10.3)	6.4 (4.4–8.5)	
Patient age, years, mean (95% CI)	60.5 (59.6–61.4)	59.1 (57.7–60.3)	61.9 (60.6–63.1)	0.001
Patient employment status				
Active	36.8 (33.4–40.1)	43.9 (39.6–48.3)	29.6 (25.7–33.5)	<0.0001
Unemployed	11.7 (9.1–14.3)	12.1 (9.0–15.1)	11.4 (8.1–14.6)	
Pensioner	42.6 (39.0–46.2)	42.8 (38.6–47.0)	42.4 (37.3–47.5)	
Housewives	8.6 (6.7–10.5)	0.7 (0.0–1.3)	16.4 (12.8–20.1)	<0.0001
Student	0.3 (0.0–0.5)	0.5 (−0.1–1.1)	0 (0.0–0.0)	
Main cause of chronic pain				
Degenerative spinal diseases	38.6 (34.4–42.8)	36.8 (32.1–41.5)	40.5 (35.2–45.9)	
Lumbosciatic pain	29.8 (26.3–33.2)	34.5 (29.9–39.0)	25.1 (20.8–29.4)	0.001
Trauma	3.1 (1.8–4.5)	3.9 (1.8–5.9)	3.9 (1.9–5.9)	
Complex regional syndrome	4.1 (2.6–5.6)	3.7 (2.1–5.3)	4.6 (2.3–6.8)	
Osteoarthritis extremities	9.0 (6.9–11.1)	6.7 (4.6–8.8)	11.2 (8.3–14.1)	0.001
Peripheral neuropathy	5.6 (4.0–7.3)	6.6 (4.3–8.8)	4.8 (2.9–6.6)	
Visceral	1.4 (0.7–2.2)	1.9 (0.8–2.9)	0.9 (0.1–1.6)	
Neoplastic	3.6 (2.4–4.8)	4.4 (2.8–6.0)	2.7 (1.3–4.1)	
Headache	0.8 (0.3–1.4)	0.3 (−0.1–0.8)	1.2 (0.3–2.1)	0.033
Fibromyalgia	4.0 (2.6–5.4)	0.5 (−0.1–1.1)	7.6 (5.1–10.2)	<0.0001
Herpes zoster	1.7 (1.0–2.4)	2.0 (0.9–3.1)	1.4 (0.4–2.3)	
Other	5.6 (3.5–7.6)	4.4 (2.8–6.0)	2.7 (1.3–4.1)	
Duration of pain, months, mean (95% CI)	29.7 (26.7–32.6)	26.9 (23.1–30.6)	32.7 (28.2–37.2)	0.007
Type of pain				
Somatic	23.2 (19.3–27.2)	19.5 (15.5–23.5)	27.2 (22.3–32.1)	0.001
Visceral	1.4 (0.7–2.1)	1.2 (0.3–2.1)	1.7 (0.7–2.7)	
Neuropathic	24.0 (20.6–27.4)	27.0 (22.9–31.2)	21.1 (17.1–25.2)	0.008
Mixed	50.2 (45.5–54.9)	52.0 (46.9–57.0)	47.9 (42.4–53.5)	
Primary	1.5 (0.5–2.6)	0.5 (−0.1–1.1)	2.6 (0.6–4.5)	0.045
Pain intensity measurement with a validated scale before referral	39.9 (31.8–47.9)	40.4 (32.1–48.8)	38.6 (30.3–46.9)	
Pain intensity (0–10) at pain unit consultation, mean (95% CI)	6.8 (6.6–6.9)	6.6 (6.4–6.8)	6.9 (6.7–7.2)	0.041
Current pain intensity (0–10 points)				
Mild (0–4)	13.1 (9.8–16.5)	15.3 (11.1–19.5)	11.1 (7.6–14.7)	0.033
Moderate (5–7)	55.5 (51.6–59.4)	56.6 (51.9–61.4)	54.7 (49.9–59.4)	
Severe (8–10)	32.5 (28.1–36.8)	29.0 (24.1–33.8)	35.6 (30.6–40.6)	0.033
Impaired functionality due to chronic pain	82.6 (78.0–87.2)	81.5 (76.6–86.4)	84.3 (79.6–89.0)	
Sleep disturbance due to chronic pain	58.7 (53.3–64.0)	56.9 (51.1–62.7)	61.7 (56.0–67.5)	
Assessment of sleep disturbance using validated scales	16.3 (10.2–22.3)	16.0 (9.7–22.2)	16.0 (9.9–22.1)	
Presence of breakthrough pain	26.6 (20.7–32.4)	26.7 (20.5–32.9)	26.2 (20.1–32.3)	
Analgesic step of the patient on visiting the pain unit				
No treatment	9.8 (7.5–12.2)	10.6 (7.8–13.4)	9.1 (6.1–12.1)	
First step: non-opioid analgesic	24.1 (20.1–28.1)	25.0 (20.1–30.0)	23.3 (18.8–27.7)	
First step: non-opioid analgesic + adjuvant	13.7 (11.5–16.0)	15.9 (12.4–19.5)	11.7 (8.7–14.7)	
Second step: weak opioid	10.8 (8.0–13.6)	9.3 (6.2–12.3)	11.6 (8.0–15.2)	
Second step: weak opioid + adjuvant	10.3 (7.9–12.7)	10.3 (7.4–13.2)	10.6 (7.4–13.8)	
Second step: weak opioid + non-opioid analgesic	8.8 (6.7–10.9)	8.0 (5.0–11.0)	9.6 (6.8–12.4)	
Second step: weak opioid + non-opioid analgesic + adjuvant	8.1 (6.0–10.2)	8.2 (5.7–10.8)	8.0 (5.3–10.8)	
Third step: strong opioid	5.5 (3.8–7.3)	5.6 (3.3–7.9)	5.7 (3.6–7.8)	
Third step: strong opioid + adjuvant	6.6 (4.7–8.5)	7.3 (4.7–9.9)	6.0 (3.7–8.2)	
Third step: strong opioid + non-opioid analgesic	4.4 (2.9–5.8)	3.4 (1.6–5.1)	5.3 (3.3–7.3)	
Third step: strong opioid + non-opioid analgesic + adjuvant	6.7 (4.5–8.9)	6.6 (4.1–9.1)	6.8 (4.1–9.6)	
Interventional techniques, drug administration via spinal route, peripheral nerve block, sympathetic or neurolytic block, electrical stimulation techniques, neurosurgery	2.2 (0.9–3.5)	2.5 (0.8–4.2)	1.8 (0.4–3.2)	
Instructions for the use of rescue analgesics	45.5 (38.4–52.5)	44.5 (37.2–51.8)	45.9 (38.4–53.3)	

CI: confidence interval.

**Table 3:** Pain treatment in 1190 patients with chronic pain referred to the pain unit.

Variables	Total % (95% CI)	Gender		*p* Value
		Men % (95% CI)	Women % (95% CI)	
Drug treatment on admission to the pain unit				
None	8.4 (5.9–11.0)	9.1 (6.2–11.9)	7.7 (4.8–10.7)	
Non-steroidal anti-inflammatory agents	37.6 (30.9–44.3)	42.6 (33.3–51.9)	34.8 (27.5–42.0)	0.049
Metamizole	24.5 (20.4–28.6)	22.0 (17.3–26.7)	28.2 (22.2–34.2)	0.042
Paracetamol	54.5 (47.8–61.1)	53.2 (45.1–61.4)	56.5 (49.2–63.9)	
Codeine	1.3 (0.4–2.2)	0.7 (−0.4–1.7)	1.8 (0.4–3.3)	
Tramadol	40.0 (34.8–45.1)	40.0 (33.4–46.7)	40.5 (34.6–46.3)	
Buprenorphine	2.5 (1.2–3.7)	2.4 (1.0–3.7)	2.8 (0.7–4.8)	
Fentanyl	9.4 (6.6–12.3)	8.3 (5.1–11.6)	10.6 (7.2–14.0)	
Hydromorphone	0.2 (−0.1–0.4)	0.2 (−0.2–0.5)	0.2 (−0.2–0.5)	
Morphine	1.6 (0.2–3.1)	1.4 (0.1–2.7)	2.0 (0.1–3.9)	
Oxycodone	0.4 (0.0–0.8)	0.5 (−0.2–1.2)	0.3 (−0.1–0.8)	
Oxycodone/naloxone	5.4 (2.8–8.1)	5.5 (3.1–7.9)	5.5 (1.7–9.2)	
Tapentadol	11.6 (8.6–14.6)	10.5 (7.1–14.0)	12.9 (8.7–17.0)	
Lidocaine	1.2 (0.4–2.0)	0.8 (0.0–1.6)	1.5 (0.3–2.6)	
Capsaicin	1.8 (0.6–3.0)	0.8 (0.1–1.6)	3.0 (0.2–5.7)	
Amitriptyline	7.4 (5.3–9.5)	6.2 (3.8–8.5)	8.5 (5.6–11.3)	
Duloxetine	1.8 (0.6–3.0)	1.0 (0.0–2.0)	2.8 (0.2–5.5)	
Venlafaxine	26.6 (22.8–30.5)	27.2 (22.1–32.4)	26.0 (21.0–30.9)	
Pregabalin	10.7 (8.3–13.1)	12.0 (8.6–15.3)	10.2 (5.9–14.4)	
Gabapentin	2.8 (1.4–4.2)	2.8 (1.1–4.4)	2.8 (1.1–4.4)	
Corticosteroids	6.5 (4.4–8.7)	7.3 (4.7–9.9)	5.6 (2.9–8.2)	
Mean drug doses on admission to the pain unit				
Metamizole (mg)	1369.1 (1223.7–1514.5)	1413.1 (1192.2–1634.0)	1329.5 (1133.0–1526.0)	
Paracetamol (mg)	1886.7 (1766.9–2006.6)	1821.0 (1645.6–1996.5)	1962.9 (1797.7–2128.1)	
Codeine (mg)	55.0 (16.0–94.0)	90.0	49.6 (5.4–93.9)	
Tramadol (mg)	139.7 (129.9–149.6)	138.7 (124.0–153.3)	140.8 (127.4–154.2)	
Buprenorphine (µg)	40.4 (32.1–48.7)	37.9 (27.2–48.6)	43.4 (28.3–58.6)	
Fentanyl (µg)	87.2 (56.5–117.8)	109.7 (52.2–167.3)	71.9 (37.8–106.0)	
Hydromorphone (mg)	8.0	-	8.0	
Morphine (mg)	35.0 (19.0–51.0)	39.0 (12.0–66.0)	32.0 (7.0–57.0)	
Oxycodone (mg)	29.0 (6.0–53.0)	24.0 (−15.0–63.0)	38.0 (−8.0–83.0)	
Oxycodone/naloxone (mg)	29.0 (22.0–36.0)	29.0 (19.0–39.0)	28.0 (17.0–40.0)	
Tapentadol (mg)	130.0 (112.0–148.0)	137.0 (105.0–170.0)	124.0 (104.0–143.0)	
Amitriptyline (mg)	24.7 (19.5–29.8)	29.1 (17.1–41.0)	22.0 (18.0–25.0)	
Duloxetine (mg)	60.0 (53.0–68.0)	53.0 (43.0–62.0)	67.0 (56.0–78.0)	
Venlafaxine (mg)	109.0 (67.0–151.0)	139.0 (10.0–267.0)	99.0 (49.0–149.0)	
Pregabalin (mg)	165.1 (148.5–181.8)	180.9 (155.7–206.1)	147.0 (126.1–167.9)	
Gabapentin (mg)	847.0 (735.0–959.0)	876.0 (737.0–1014.0)	805.0 (608.0–1002.0)	
Actions taken at the pain unit regarding drug treatment				
No action, previous treatment maintained	11.9 (8.8–15.1)	13.3 (9.7–16.8)	10.6 (7.1–14.1)	
First step dose modification	10.4 (5.8–14.9)	10.5 (6.1–14.9)	11.2 (4.8–17.6)	
Second step dose modification	14.6 (10.3–18.8)	14.3 (10.0–18.5)	13.8 (9.0–18.7)	
Third step dose modification	10.4 (6.9–13.8)	8.4 (5.2–11.6)	12.4 (6.7–18.1)	
Change of the first step non-opioid analgesic	7.7 (3.5–11.8)	6.1 (2.8–9.4)	10.0 (2.6–17.3)	
Moved from the first to the second step	16.8 (12.4–21.3)	17.6 (12.8–22.4)	16.9 (10.7–23.2)	
Change-rotation of second step opioid	4.7 (1.4–8.1)	4.3 (1.5–7.1)	6.0 (0.5–11.5)	
Change weak to strong opioid (2nd to 3rd step)	16.2 (11.2–21.2)	14.9 (10.6–19.2)	18.6 (11.0–26.2)	
Change-rotation of third step opioid	6.7 (3.1–10.3)	7.2 (4.3–10.1)	7.2 (0.3–14.1)	
Change of adjuvant	7.8 (4.3–11.4)	6.4 (3.4–9.4)	10.2 (4.4–16.0)	
Addition of one or more non-opioid analgesics	7.5 (5.0–10.1)	7.8 (4.8–10.9)	7.4 (4.5–10.3)	
Addition of one or more adjuvants	28.1 (23.3–33.0)	28.0 (22.5–33.5)	28.3 (22.6–34.0)	
Start of interventional techniques, drug administration via spinal route, peripheral nerve block, sympathetic or neurolytic block, electrical stimulation techniques or neurosurgery	60.4 (44.6–76.3)	60.0 (48.1–71.9)	53.0 (44.1–61.9)	
Reasons for treatment changes				
Lack of efficacy	60.2 (55.2–65.1)	62.1 (56.6–67.6)	58.4 (64.1)	
Side effects	9.1 (6.7–11.4)	7.8 (5.3–10.3)	10.3 (7.3–13.4)	
Insufficient dose	24.2 (19.6–28.7)	23.6 (18.8–28.4)	25.1 (19.8–30.4)	
Others	10.6 (7.5–13.8)	13.6 (6.5–20.8)	9.8 (6.5–13.2)	
Patient follow-up				
Refer the patient for a second consultation appointment at the pain unit	90.3 (87.9–92.8)	90.8 (87.8–93.7)	89.8 (86.8–92.9)	
Refer the patient to the service from which he/she was referred	2.8 (1.5–4.1)	2.2 (1.0–3.4)	3.4 (1.5–5.3)	
Refer the patient to a different specialist from which he/she came from	2.6 (1.4–3.8)	2.4 (0.9–3.9)	2.9 (1.4–4.3)	
Refer the patient to primary care	4.6 (2.9–6.4)	5.2 (3.1–7.3)	4.1 (2.1–6.0)	
Modification of the previous diagnosis	17.3 (13.1–21.5)	14.8 (10.6–19.0)	19.0 (14.4–23.6)	0.043
Exclusion of addictive disorders in opioid-treated patients	57.6 (49.7–65.4)	60.6 (52.4–68.8)	55.2 (47.0–63.5)	
Information that opioids may affect driving ability	59.3 (51.5–67.0)	61.6 (53.5–69.8)	57.9 (49.5–66.4)	

CI: confidence interval.

## Data Availability

Data of the study are available from the corresponding author upon request.

## Supplementary Materials

The following supporting information can be downloaded at: https://www.mdpi.com/article/10.3390/jcm11133586/s1, Study questionnaire. The Team DUO (members by alphabetical order): Carolina Abellán Aracil, Aranjuez, Madrid; Alejandro Alcina Navarro, Madrid; Ignacio Javier Alejandro Alejandro, Las Palmas; Lucía Ángel Redondo, Sevilla; Mónica Araujo Vázquez, Pontevedra; Martín Zacarías Arcas Molina, Albacete; Enrique Bárez Hernández, Araba/Álava; Dolores Bedmar Cruz, Madrid; Moncef Belaouchi Belaouchi, Valencia; Ara Bermejo Marín, Valencia; José David Bravo Corrales, Ciudad Real; Carlos Burguera Baldoví, Valencia; José Enrique Calderón Seoane, Cádiz; Noelia Calvo García, Bizkaia; Elena Cano Serrano, Granada; Luz Cánovas Martínez, Ourense; Miguel Ángel Caramés Álvarez, Las Palmas; Martín Carpintero Porrero, Asturias; Félix Cebeiro Balda, Navarra; Joan Coma Coma, Barcelona; José de la Cueva Aguilera Cádiz; Juan Carlos de la Pinta García, Madrid; Marta del Valle Hoyos, Málaga; Eva María Díaz Ibáñez, Murcia; Clara Díaz-Alejo Marchante, Alicante; Enrique Domínguez Suárez, Lugo; Francisco Duca Rezzulini, Barcelona; Cristina Embid Román, Alicante; Sara Estévez Sarmiento, Las Palmas; Gustavo Fabregat Cid, Valencia; Jaime Fandiño Vaquero, Pontevedra; Mariano Fernández Baena, Málaga; Alfredo Fernández Esplá, Madrid; María del Rosario Fernández Fernández, Asturias; Montserrat Fernández García, Barcelona; Ana Fernández López, Madrid; Urides Armando Fernández Orraca, Barcelona; Lourdes Ferreira Laso, La Rioja; Julia M. Ferreras Zamora, Barcelona; Jordi Folch Ibáñez, Barcelona; Cristina Garcés San José, Zaragoza; Juan Carlos García Collada, Cuenca; María Jesús García Menéndez, Asturias; José Antonio Girón Mombiela, Zaragoza; José Luis Gómez Palonés, Castellón; María Cristina Gómez Vega, Bizkaia; Juana González Fernández, Granada; Eduardo González García, Valladolid; Raquel González Jiménez, Madrid; José Manuel González Mesa, Málaga; Marcos González Cabano, A Coruña; Agustín Guerri Cebollada, Valencia; Jordi Guitart Vela, Barcelona; Mª Anunciación Gutiérrez Gómez, Burgos; Zeina Hachoue Saliba, Madrid; María José Hernandez Cádiz, Valencia; María Inmaculada Herrador Montiel, Córdoba; Carla Iglesias Morales, Toledo; Rosa Ma Izquierdo Aguirre, Valencia; Paula Jiménez Vázquez, Sevilla; Ma Rocío López Díez, Ourense; Josep López Garrido, Barcelona; María López Gómez, Toledo; Mónica López-Tafall Cáceres, Araba/Álava; José Leonardo Mansilla Moret, IIIes Balears; Jenaro Mañero Rey, Barcelona; César Margarit Ferri, Alicante; Diana Mariñansky Mlynarzewicz, Girona; Mercedes Martínez García, Madrid; Javier Mata Estévez, IIIes Balears; Mónica Mayo Moldes, Pontevedra; Javier Medel Rebollo, Barcelona; Ana Belén Mencías Hurtado, Santa Cruz de Tenerife; Juan Manuel Mercado Escribá, Castellón; Bartolomé Mir Darder, IIIes Balears; Rosse Mery Molina Sánchez, Barcelona; María del Mar Monerris Tabasco, Barcelona; Onel Morales Torres, Girona; Rafael Morales Valero, Córdoba; Manuel Muñoz Martínez, Madrid; María Navarro Martínez, Burgos; Minerva Navarro Rivero, Las Palmas; María Luz Padilla Del Rey, Murcia; Ángela Catalina Palacios Córdoba, Granada; Ernesto Pastor Martínez, Valencia; María Amparo Pérez Díaz, A Coruña; Arelis Pérez Fernández, Albacete; Guillermo Petinal Algás, Pontevedra; Mauricio Polanco García, Barcelona; Patricia Puiggròs Hernández, IIIes Balears; Miriam Puyo Olmo, Barcelona; Montserrat Reche García, Valencia; Ma Carmen Ribera Montés, Alicante; Paloma Ricós Bugeda, Barcelona; María Belén Rodríguez Campoó, Madrid; Isabel Rodríguez Fernández, Cáceres; Iria Rodríguez Rodríguez, A Coruña; María Cristina Rodríguez Roca, Madrid; Elena Rojo Rodríguez, Madrid; María Blanca Rondeau Marco, Murcia; Alberto Sánchez Campos, Bizkaia; Esperanza Sánchez Navarro, Madrid; Calixto Andrés Sánchez Pérez, Alicante; Roger Sánchez Toll, Valencia; José Antonio Sánchez Tirado, Zaragoza; Yolanda Sastre Peris, Alicante; Ancor Serrano Afonso, Barcelona; Anna Server Salvà, Barcelona; Carlos Solano Perea, Cádiz; Ana Suárez Cobian, Madrid; Alberto Taborga Echevarría, Cantabria; Humberto Víctor Torres Lamberti, Asturias; Ma Isabel Vargas Domingo, Barcelona; Alberto Vela de Toro, Granada; Rafael Vergel Eleuterio, Murcia; Vicente Luis Villanueva Pérez, Valencia; Francisco Villegas Estévez, Castellón; and Marta Yus López, Madrid, Spain.
