# Supplementary material for: Assessment of the Management of Patients with Chronic Pain Referred to a Specialized Pain Unit: A Cross-Sectional Multicenter Study (the DUO Project)
Source: J Clin Med. 2022 Jun 22;11(13):3586. doi: 10.3390/jcm11133586 (PMC9267154; doi:10.3390/jcm11133586)
Supplement: Supplementary file 1 [file jcm-11-03586-s001.zip › jcm-1743030-supplementary.pdf]

Questions asked to assess the structure and process indicators in the care of patients with chronic pain

**Indicators of the participating physician**		
1. Gender of the participating physician	☐ Male<br>☐ Female	
2. Age of the participating physician		
3. Years of professional experience in the field of pain (excluding years of resident training) years)		
4. Current clinical specialty	☐ Anesthesia<br>☐ Internal Medicine (general practitioner)<br>☐ Primary care<br>☐ Others; specify	
**Structure indicators**		
5. Type of center to which the unit belongs	☐ Public hospital/center<br>☐ Private hospital/center<br>☐ Mixed center	
6. What level does your pain unit correspond to?	☐ Level I<br>☐ Level II<br>☐ Level III	
7. Number of patients seen in the unit in one month		
8. Number of new patients referred to the unit in one month		
9. Does the unit have a pain assessment protocol?	☐ No<br>☐ Yes	Availability of an action protocol for the assessment of chronic pain in the pain unit
10. Does the unit use validated chronic pain assessment scales?	☐ No<br>☐ Yes	Use of validated scales for the assessment of chronic pain in the pain unit
11. Is health-related quality of life of patients with chronic pain evaluated using validated scales?	☐ No<br>☐ Yes	Use of scales to assess health-related quality of life in the pain unit
12. Is mental health of patients with chronic pain evaluated using validated scales?	☐ No<br>☐ Yes	Assessment of patient mental health using validated scales in the pain unit
**Process indicators**		
1. From which specialty is the patient referred?	Orthopaedic surgery and traumatology<br>Neurology/neurosurgery<br>Rheumatology<br>Primary care<br>Oncology<br>Physical medicine and rehabilitation<br>Internal medicine<br>Others	Origin of the patient referred to the pain unit for te first time
2. Age		
3. Patient employment status	Active<br>Unemployed<br>Pensioner<br>Housewives<br>Student	
4. Indicate the main cause of chronic pain	Degenerative spinal diseases<br>Lumbosciatic pain<br>Trauma<br>Complex regional syndrome<br>Osteoarthritis extremities<br>Peripheral neuropathy<br>Visceral<br>Neoplastic<br>Headache<br>Fibromyalgia<br>Herpes zoster<br>Other	Proportion of patients referred to the pain unit according to the main cause of chronic pain
5. How long has the patients suffered from chronic pain (months)?		Time from onset of chronic pain to referral to the pain unit

Level I: monographic unit; Level II: unidisciplinary pain treatment unit; Level III: multidisciplinary unit for the study and treatment of pain.

Questions asked to assess the structure and process indicators in the care of patients with chronic pain (continued)

**Process indicators**		
6. Indicate the type of pain the patient has	Somatic<br>Visceral<br>Neuropathic<br>Mixed<br>Primary	
7. Has the pain intensity being assessed using some validated scale before referral to the pain unit?		Assessment of pain intensity using validated scales in the specialties from which the patient has been referred
8. On a 0-10 point scale, what is the patient pain intensity score at the time of the visit?		Pain intensity at the time of the visit as an evaluation of the degree of chronic pain control prior to referral to the pain unit.
9. Indicate the current patient pain intensity	Mild (0-4 points)<br>Moderate (5-7 points)<br>Severe (8-10 points)	Pain intensity at the time of the visit as an evaluation of the degree of chronic pain control prior to referral to the pain unit
10. Is the patient functionally affected by the chronic pain?		Proportion of patients with impaired functionality due to chronic pain
11. Is the patient sleep affected by the chronic pain?		Proportion of patients in whom sleep is affected due to chronic pain
12. Was a validated scale used to assess patient sleep disturbance due to chronic pain?		Proportion of patients assessed for sleep disturbance due to chronic pain
13. Does the patient suffer associated breakthrough pain?		Proportion of patients with chronic pain who suffer breakthrough pain
14. What is the analgesic step of the patient on visiting the unit for the first time?	– No treatment<br>– First step: non-opioid analgesic<br>– First step: non-opioid analgesic + adjuvant<br>– Second step: weak opioid<br>– Second step: weak opioid + adjuvant<br>– Second step: weak opioid + non-opioid analgesic<br>– Second step: weak opioid + non-opioid analgesic + adjuvant<br>– Third step: strong opioid<br>– Third step: strong opioid + adjuvant<br>– Third step: strong opioid + non-opioid analgesic<br>– Third step: strong opioid + non-opioid analgesic + adjuvant<br>– Interventional techniques, drug administration via spinal route, peripheral nerve block, sympathetic or neurolytic block, electrical stimulation techniques, neurosurgery	Proportion of patients not receiving treatment for chronic pain at the time of referral to the pain unit<br><br>Proportion of patients in each step and combined treatments of the WHO analgesic ladder
15. Was the patient instructed on the use of rescue analgesia when pain persists despite prescribed treatment?		Proportion of patients instructed on the use of rescue analgesia when pain persists despite prescribed treatment, prior to referral to the pain unit

Questions asked to assess the structure and process indicators in the care of patients with chronic pain (continued)

**Process indicators**		
16. Indicate below the chronic pain treatments received by the patient at the time of admission to the pain unit (one or more drugs may be indicated)	– None<br><br>– Non-steroidal anti-inflammatory drugs<br>– Metamizole<br>– Paracetamol<br>– Codeine<br>– Tramadol<br>– Buprenorphine<br>– Fentanyl<br>– Hydromorphone<br>– Morphine<br>– Oxycodone<br>– Oxycodone/naloxone<br>– Tapentadol<br>– Lidocaine<br>– Capsaicin<br>– Amitriptyline<br>– Duloxetine<br>– Venlafaxine<br>– Pregabalin<br>– Gabapentin<br>– Corticosteroids<br>– Another adjuvant agent	Proportion of patients not receiving treatment for chronic pain at the time of referral to the pain unit<br><br>Proportion of patients receiving treatment for chronic pain with each drug substance, prior to referral to the pain unit
17. Indicate below the total daily dose of the chronic pain treatment received by the patient and the time of admission to the pain unit (one or more drugs for one same patient may be considered)	– Metamizole<br>– Paracetamol<br>– Codeine<br>– Tramadol<br>– Buprenorphine<br>– Fentanyl<br>– Hydromorphone<br>– Morphine<br>– Oxycodone<br>– Oxycodone/naloxone<br>– Tapentadol<br>– Amitriptyline<br>– Duloxetine<br>– Venlafaxine<br>– Pregabalin<br>– Gabapentin	Mean doses of each drug administered to patients at the time of admission to the pain unit
18. Indicate the action taken regarding patient treatment at the end of the first visit to the pain unit (one or more options)	– None, previous treatment maintained<br>– Modified the dose of the first step<br>– Modified the dose of the second step<br>– Modified the dose of the third step<br>– Change of the first step non-opioid analgesic<br>– Moved from the first to the second step<br>– Change-rotation of second step opioid<br>– Change from weak opioid to strong opioid (2nd to 3rd step)<br>– Change-rotation of third step opioid<br>– Change of adjuvant<br>– Addition of ≥ 1 non-opioid analgesics<br>– Addition of ≥ 1 adjuvants<br>– Start of interventional techniques, drug administration via spinal route, peripheral nerve block, sympathetic or neurolytic block, electrical stimulation techniques or neurosurgery	Proportion of patients with some change of treatment for chronic pain in the pain unit<br><br>Proportion of patients subjected to each treatment modifying action prior to referral to the pain unit
19. The treatment changes were mainly due to:	Lack of efficacy<br>Side effects<br>Insufficient dose<br>Others	Proportion of patients with some change in prior treatment for chronic pain according to each main reason

Questions asked to assess the structure and process indicators in the care of patients with chronic pain (continued)

**Process indicators**		**Indicator**
20. Indicate how patient follow-up was made	– Patient appointment for second visit to the pain unit<br>– Patient sent to the service from which he/she was referred<br>– Patient sent to a specialty different from which he/she was referred<br>– Sent to primary care	Proportion of patients subjected to each referral action after the first visit to the pain unit
21. Was a change made in the diagnosis with which the patient was referred?		Proportion of patients with a change in prior diagnosis before the first visit to the pain unit
22. If an opioid was prescribed, were addictive disorders discarded before starting opioid treatment?		Proportion of patients prescribed an opioid in which addictive disorders were discarded before starting treatment with opioids in the pain unit
23. If an opioid was prescribed, was the patient informed that it may affect his/her ability to drive, especially at the start of treatment, after an increase in dose, or after switching from another medication?		Proportion of patients prescribed an opioid in which the dose was increased who were informed that it may affect his/her ability to drive, especially at the start of treatment
